# RBEF: Ransomware Efficient Public Blockchain Framework for Digital Healthcare Application

**DOI:** 10.3390/s23115256

**Published:** 2023-06-01

**Authors:** Abdullah Lakhan, Orawit Thinnukool, Tor Morten Groenli, Pattaraporn Khuwuthyakorn

**Affiliations:** 1 School of Economics, Innovation and Technology, and Kristiania University College, 0107 Oslo, Norway; 2 Embedded System and Computational Science, College of Arts, Media and Technology, Chiang Mai University, Chiang Mai 50200, Thailand; 3 RSISE, Australian National University, Canberra, ACT 0200, Australia; 4 College of Arts and Technology, Chiang Mai University, Chiang Mai 50200, Thailand

**Keywords:** blockchain, RBEF, ransomware, delays, sandbox, static and dynamic analysis

## Abstract

These days, the use of digital healthcare has been growing in practice. Getting remote healthcare services without going to the hospital for essential checkups and reports is easy. It is a cost-saving and time-saving process. However, digital healthcare systems are suffering from security and cyberattacks in practice. Blockchain technology is a promising technology that can process valid and secure remote healthcare data among different clinics. However, ransomware attacks are still complex holes in blockchain technology and prevent many healthcare data transactions during the process on the network. The study presents the new ransomware blockchain efficient framework (RBEF) for digital networks, which can identify transaction ransomware attacks. The objective is to minimize transaction delays and processing costs during ransomware attack detection and processing. The RBEF is designed based on Kotlin, Android, Java, and socket programming on the remote process call. RBEF integrated the cuckoo sandbox static and dynamic analysis application programming interface (API) to handle compile-time and runtime ransomware attacks in digital healthcare networks. Therefore, code-, data-, and service-level ransomware attacks are to be detected in blockchain technology (RBEF). The simulation results show that the RBEF minimizes transaction delays between 4 and 10 min and processing costs by 10% for healthcare data compared to existing public and ransomware efficient blockchain technologies healthcare systems.

## 1. Introduction

Cyberattacks regarding ransomware on healthcare institutions have been pinpointed recently, and it is an exceptional threat to healthcare data. The University of Vermont (UVM) Medical Center published an article on ransomware attacks on hospital systems [1]. UVM claimed that many patients complain to hospital staff about strange computer data accessibility problems for online access to the hospital. Even though UVM employees could not access their payroll system, patient appointments were automatically rescheduled. The attacked data were converted into an encrypted form, and now they need to pay about 30 million US dollars to decrypt it with distinct keys. The blockchain is an efficient technology to process and validate the data in healthcare systems. However, many attacks have recently been applied to the blockchain to reverse the original operation with a false one. In blockchain-enabled healthcare, the number of ransomware attacks has been going up over time. Blockchain technology is the decentralized paradigm where all transactions are processed in an immutable, valid, and transparent form among nodes. Ransomware attacks are adaptive and prevent the transaction from being performed on the blockchain frameworks [2]. Generally, blockchain-enabled healthcare applications, such as the heartbeat monitoring system, blood pressure real-time monitoring system, online remote counseling, and self-adaptive report checking, are distributed and connected to the many healthcare services that are deployed at different hospitals. Users can store and install healthcare apps on their mobile devices and then send the data of the application to the hospital so the system can be analyzed and run. The main goal is to let applications run on the server so that processing time and energy use are kept to a minimum, and so that dynamic real-time healthcare applications can use as many resources as they need. The main issue is the data security during offloading and execution on the server via base stations and different wireless technologies in the system. The patient’s data are offloaded to the cloud servers for performance via other base stations. Therefore, security is a significant risk in the mobile cloud environment for execution [3].

Many security blockchain-enabled systems [4,5,6,7,8,9,10] are suggested for distributed healthcare application in mobile cloud networks. The goal is to reduce the security risk during computational offloading from mobile devices to the cloud server for execution. Mobile cloud healthcare applications are those in which small workloads are executed on mobile devices, and compute-intensive workloads are offloaded to the cloud for performance. These studies [11,12,13,14,15] focused on the decentralized blockchain security model where users offload tasks to the cloud for the computation and security aspects of the node.

Many challenges that exist in prior blockchain technologies are suggested for healthcare application: (i) It is acceptable, meaning the blockchain is an efficient and secure technology, but ransomware attacks degraded the performance of the scheduled workload due to long transaction delays. (ii) In all of the previous studies, blockchain technology was used to meet the security needs of distributed healthcare applications. However, blockchain is a resource-consuming technology and incurs longer delays during the processing of healthcare workloads.

### Motivation and Methodology

In this paper, we present efficient security mechanisms based on blockchain technology and Cuckoo Sandbox static and dynamic schemes. The digital healthcare application is distributive, where a few healthcare tasks are executed locally, and the rest of them are executed on the server. Therefore, for security reasons, we combined the blockchain validation scheme with the sandbox scheme to handle both runtime and compile-time ransomware attacks. However, existing blockchain technologies for healthcare applications are very time-consuming and incur higher delays. Therefore, we optimize the transactional delays and processing costs of healthcare tasks on mobile fog and cloud nodes. The paper makes the following contributions:

1. This paper introduces the RBEF blockchain simulator, which was developed using Android and Kotlin languages, with the aim of incorporating hospitals, wireless, and other healthcare services into the framework for execution;
2. The RBEF simulator consists of static, dynamic and blockchain validation schemes. All nodes (e.g., mobile, wireless network, fog and cloud computing) perform blockchain hashing, and smart-contract rules validate all transactions against ransomware attacks and their encryption within the framework;
3. A mathematical model is presented for the problem under consideration, based on different constraints such as delay, security, and resources required for processing blockchain validation against malware in healthcare applications;
4. We implemented the cuckoo sandbox static and dynamic data sampling schemes to validate the blockchain transaction among nodes. We handle both static and dynamic ransomware attacks based on the pattern of transactions among nodes.

This paper is organized as follows: Section 2 discusses the related work concerning blockchain technology and ransomware attacks on healthcare data. Section 3 presents the blockchain ransomware framework, while Section 4 describes the algorithm and steps involved. Section 5 showcases the simulation implementation with the different configuration. Section 6 shows the results discussion with the existing approaches and proposed work. Section 7 concludes the paper by discussing the achieved results and outlining future work.

## 2. Related Work

These days, digital healthcare technologies are based on mobile, wireless, and fog cloud, which have been gaining a lot of attention. Many existing digital healthcare systems are integrated with different schemes, protocols, applications, and objectives for remote healthcare services. The primary objective is to offer digital healthcare services securely via various healthcare applications.

Table 1 shows the existing efforts of studies for healthcare applications based on blockchain technologies.

One such study [1] introduces a hyper ledger blockchain-enabled application to offload and download secure remote healthcare data on mobile and cloud networks. The Hyper Text Transfer Protocol (HTTP) and File Transfer Protocol (FTP) are communication protocols used for offloading and downloading healthcare data among blockchain nodes. This study only focused on known malware and ransomware detection. The proposed work has a higher processing delay.

In another study [2], the authors presented a proof-of-work (PoW) validation based on static analysis for ransomware attack detection. The study integrated a digital healthcare system based on client–server architecture. The blockchain PoW validation was applied only to the server nodes due to the availability of high resources. The static analysis ransomware methodology was implemented, where encrypted data patterns were annotated at the header of each file. Therefore, criminal activity can easily be determined from the header pattern of data during transactions among nodes. The mobile devices were utilized as thin clients and only downloaded healthcare data from servers. However, overall processing incurred higher delays.

These studies [3,4,5] suggest proof of credibility (PoC) blockchain transaction validation schemes and static analysis techniques for ransomware detections. The studies consider the transmission control protocol (TCP) and internet protocol (IP) for requesting and responding between client and server in blockchain-based healthcare architecture. The client layer is considered to be the thin layer where the client can only offload and download data. The server layer is the key layer, where a number of heterogeneous nodes process the healthcare data among each other based on blockchain validation. Each blockchain block has a discriminative size and encrypts and decrypts data based on the SHA-256 algorithm. The static analysis technique of the ransomware attacks was implemented in distributed healthcare. Each blockchain block transferring the data was based on designated pattern of files with the X86 operating system. Therefore, the extension of files is fixed. If the pattern slightly changed and its replica copied on different blocks, it was identified as ransomware attack in the healthcare network. However, processing delays, costs, and deadlines are not considered for healthcare services and tasks in these works. 

Other studies [6,7,8] present blockchain technologies based on a semantic analysis scheme. The objective was to detect anonymous cyberattacks on healthcare networks. These studies examine healthcare transactions in uniform, heterogeneous mobile, and cloud computing environments. Each blockchain block has properties, such as hashing, transaction data, block ID, Merkle, predicate logic, and relationships. Each blockchain block can validate the data transactions based on predicated logic and rules based on static analysis. These studies analyzed the attacks based on hashing patterns, heuristic logic, predicated relationships, and relationships during data transactions among nodes. However, these methods result in higher resource consumption, longer processing times, and higher storage costs for healthcare data.

Further studies [9,10,11,12,13,14,15] suggest file and code analysis based on blockchain ransomware attack efficiency methods. With the advancement in security based on the blockchain mechanism, the failure of transactions due to cyberattacks will be a future topic in these studies. At the start of the 21st century, associated research institutes took an impressive initiative to launch HTML. That initiative made secure blockchain-based consensus strategies for communication technology evident and entered a growing technological period. The dynamic attacks enhance its capability to make it flexible and secure and prompt connectivity between on-premise devices, which will include humans; physical and other medical assets are also included. With the growing need of security in communication that is based on sockets programming, the fixed type of malware security features during passing data from one point to another is handled by suggested blockchain systems in these studies. The user datagram protocol (UDP) and TCP/IP carry the header data of the blockchain blocks with the pre-designed pattern and behavioral in each file. The data have only a fixed form, and static analysis ransomware tools identified the ransomware pattern and behavior in the network based on pre-trained sample data in the healthcare network. However, these techniques suffer from resource leakage, network consumption, processing delays, and processing cost. These blockchain healthcare technologies for the healthcare applications have a limited number of services and tasks with the fixed healthcare nodes.

These studies [16,17,18,19,20,21,22] present the security of blockchain and artificial intelligence (AI)-empowered competent healthcare and distributed dynamic mutual identity authentication for referral-based healthcare systems. The objective is to detect ransomware at-tacks at runtime after the execution of files among nodes. The suggested blockchain architectures are hybrid and in a consortium to support the media file of healthcare records in homogeneous and heterogeneous healthcare nodes. Online and offline blockchain policies were implemented in these studies. The primary constraints, such as transactional delays, are minimized in some instants and trained with ransomware sampling based on AI algorithms. However, in these studies, data privacy on mobile devices is largely disregarded, and only the main consensus blockchain facilitates the data training and operation. It shows that the mobile patient should share all data with the cloud node in terms of data through TCP protocols. However, there are many limitations to these methods. It is a server-based application, and client devices only support a limited range of healthcare services.

These studies [22,23,24,25] present the dynamic analysis of ransomware and malware sandboxing techniques for blockchain-enabled applications. The objective is to identify suspicious files with their extensions while processing healthcare services in blockchain-based networks. The healthcare applications are designed for Android devices, where the X86 operating system runs both the client and server sides for the same applications. They integrated the dynamic analyzer into the secure socket layer and transport layer security during offloading and downloading of patient data between mobile and cloud servers. However, the sandbox dynamic analyzer API only exploited data travel on the network layer. Therefore, blockchain technology suffers from resource leakage and higher processing delays in mobile healthcare applications. Lastly, studies [26,27,28,29,30,31] suggest the blockchain validation scheme and infected virtualized nodes have volatility and processing memory granularity, employing the YARA framework. The virtualized infected files can degrade the added node’s performance as the transaction failure ratio increases. Therefore, healthcare tasks and services with deadlines suffered a lot due to the time-consuming processing of new network consensus blocks. 

To our knowledge, a dynamic and static ransomware analysis for healthcare tasks and services in blockchain-enabled digital healthcare systems has yet to be conducted. Existing studies did not consider these constraints, such as transactional delay and costs, along with the deadline for each application transaction on the blockchain. Therefore, we present RBEF, which optimizes dynamic and static ransomware attack-efficient blockchain technology for digital healthcare. Our goal is to execute all healthcare transactions without any security breach and optimize transaction delay, cost, and deadlines for healthcare applications.

## 3. Proposed Architecture

**Figure 1:**
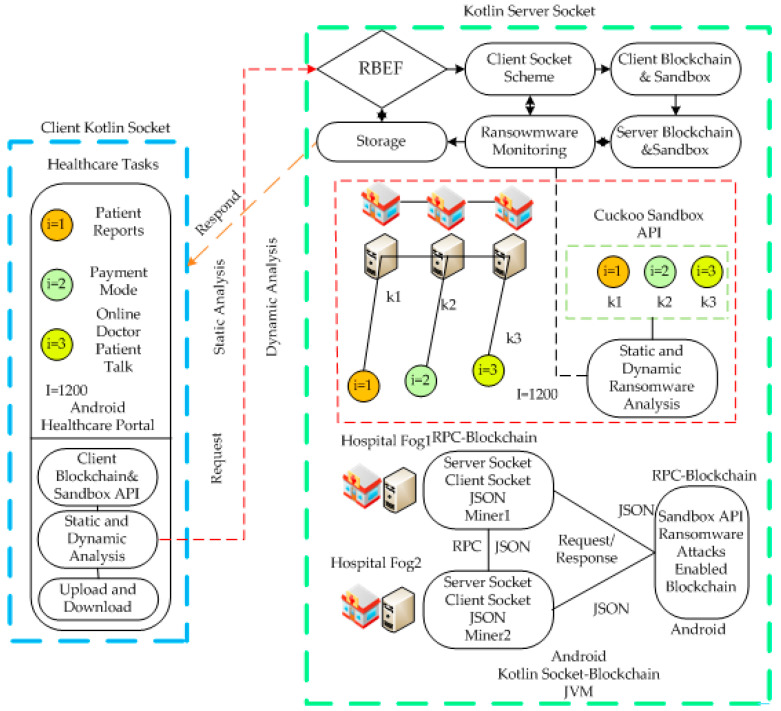
Ransomware Efficient Blockchain-Enabled Architecture for Healthcare Application.

The study presents the architecture that consists of different parts, as shown in Figure 1. The system has three socket layers: client, wireless, and fog cloud. The client socket is the thin client that installs and initiates applications based on the blockchain. The socket client’s blockchain manager consists of the following components. For instance, the Workload Offloader Engine, Network Status, Service Status, and Results. The client socket considers the different coarse-grained healthcare tasks, i, such as {i=1,2,…,I}. Each healthcare must be encrypted and decrypted in the blockchain process and offloaded to the wireless based on the system’s network and service status. We implemented the cuckoo sandbox API based on dynamic and static analysis of the ransomware scheme at the client and server socket sides. Our purpose is to detect ransomware during and before execution if it occurs during data transactions in a client–server-based healthcare system. We check the status of available nodes at real-time in terms of resource availability and security status of transactions. Each healthcare task has a deadline, hashing, and validation attributes. Whereas client sockets have resource constraint issues in their design, blockchain processes are only done locally. The workload off loader engine offloads the workload to the base station socket for processing. In contrast, each base station validated the blockchain hashing based on the proof of validation method in the system. If the previous and current hashes match, it will not offload additional workload to the fog cloud for processing.

The server socket consisted of fog and cloud nodes for healthcare tasks. In RBEF, the algorithm methodology consisted of a client socket scheme, a client socket blockchain, a cuckoo sandbox scheme, a server blockchain sandbox scheme, and a ransomware-aware strategy. The client-side socket offloads all tasks to the server socket for execution based on blockchain validation rules. For instance, tasks could include login data, registration data, medical reports, online communication, etc. Therefore, each node is responsible for following the blockchain validation process, where data are encrypted and decrypted based on the asymmetric SHA-256 scheme. Our architecture connects local fog and global cloud hospitals and share their data based on validation rules. The architecture trained each node based on static and dynamic sandbox schemes to avoid ransomware. Each blockchain transaction must validate the data based on preset patterns, and nodes can understand the pattern and behavior of the data during transactions among nodes. Generally, the socket client–server architecture is integrated with the remote procedure call (RPC) mechanism. The client sends data to the server in JavaScript object notation (JSON) form with blockchain and efficient ransomware patterns. The client and server run on the Java virtual machine (JVM) and Delvik virtual machine (DVM).

The primary agent responsible for scheduling workloads for processing is the fogging agent, which allocates workloads to either the fog node or cloud computing resources. The fog node agent transmits data between nodes in the JSON format, allowing clients and servers to exchange and communicate data based on TCP/IP protocols. Workloads are scheduled in the system using the knapsack method, where space and resources are the basis for scheduling all workloads.

The proposed framework has different steps. In the first step, all the workloads are analyzed on the local devices. Before offloading to the server for the processing, the smart contract-based regulation converts all requested data into an encryption based on advance standard encryption (AES). The ransomware engine is the method that verifies the cipher data and generates the status of the data, which have no ransomware attacks at the client side. The client side is Kotlin based on the client socket, where all the blockchain transactions of the workloads offloaded to the server socket based on Kotlin for the processing. All the requested cipher workloads, such as I=1,2,3, are scheduled to the heterogeneous workloads, such as k=1,…,K.

### Problem Formulation

The problem formulation shows how to calculate the local processing delay, base station processing delay, fog and cloud processing delay, and processing cost of the healthcare tasks. Table 2 shows the notations of formulation for the considered problem.

The study considers the I number of workloads in the system, e.g., {i=1,...,I}. The study has healthcare task i with the different attributes, $w_{i}$, $d_{i}$, as theworkload and deadline, respectively. The study considered the K number of computing nodes,{k=1,...,K} such as fog computing and cloud computing nodes with their distinct speed, e.g., ζk. We considered the mobile devices as the local client, e.g., {m=1,...M}. Each mobile device m has a distinct speed, e.g., ζm.

The study considered the B number of base stations, {b=1,…,B}. The study makes the binary assignment of the workloads on the nodes in the following way.

(1) $$x\left\{i,k\right\}=\left\{0,1,2,3\right\}$$

In Equation (1), *x*{*i,k*} = 1 shows the workload execution on the local machine, *x*{*i,k*} = 2 shows the workload executed on the fog node, and *x*{*i,k*} = 3 shows the cloud computing execution. On the other hand, *x*{*i,k*} = 0 shows the workload that is not assigned yet. The base station assignment done in the following way.

(2) $$y\left\{i,b\right\}=\left\{0,1\right\}$$

Equation (2) shows the either workload assigned to the base station or not during offloading from mobile device to the fog cloud for the processing. Whereas $y\left\{i,b\right\}=1$ denotes the workload assigned to the base station, otherwise it is equal to $y\left\{i,b\right\}=0.$ In our proposal, the workload is executed on the different nodes such as mobile device, fog and cloud nodes. The main reason is that workload is compute-intensive and requires a lot of resource for the execution. In our case, we only applied the blockchain validation based on proof of validation (PoV) and offload the data to the base station. The local delay is determined at the local machines in the following:

(3) $$L_{i}^{e}=\sum_{m=1}^{M}\;x\left\{i,m\right\}\frac{i}{\zeta m}+bct$$

Equation (3) determines local execution delay on the mobile device, whereas bct is the blockchain process time that must be added with the data transaction validation, which is determined in the following way.

(4) $$bct=\sum_{bc=1}^{BC}i\sim encryption+decryption+validation,i=1,..,I$$

Equation (4) shows the encryption time and decryption of the blockchain for all tasks in the system. The encryption time is determined in the following.

(5) $$encyption=SHA-256\sim PK\left(i\right),i=1,..,I$$

Equation (5) determines the encryption time of the workload with the *SHA*-256 with the 256-bit public key in the system.

(6) $$decyption=SHA-256\sim PV\left(i\right),i=1,..,I$$

Equation (6) determines the encryption time of the workload with the *SHA*-256 with the 256-bit public key in the system.

(7) Validation=k1,b1,b,B,K,i=1,..,I

Equation (7) determines offloading processing time determined in the following equation. The upload bandwidth could be varied during change locations in the network.

(8) $$T_{i}^{e}=\sum_{b=1}^{B}\frac{wi}{Upload-bandwidth}+bct$$

Equation (8) determines the communication time of tasks during downloading. The upload bandwidth could be varied during change locations in the network, whereas bct is the blockchain process time, which must be added with the data transaction validation.

(9) $$b_{i}^{e}=\sum_{b=1}^{B}\frac{wi}{dow-bandwidth}+bct$$

Equation (9) determines the fog node computation time and blockchain time in the system, whereas bct is the blockchain process time, which must be added with the data transaction validation.

(10) $$f_{i}^{e}=\sum_{k=1}^{K}\frac{wi}{\zeta k}+bct$$

Equation (10) determines the transactional cost of all tasks, whereas bct is the blockchain process time, which must be added with the data transaction validation.

(11) $$c_{i}^{e}=\sum_{k=1}^{K}\sum_{b=1}^{B}\sum_{M=1}^{M}\frac{wi}{\zeta k}+\frac{wi}{\zeta m}+bct$$

Equation (11) determines the cloud execution delay, including in the system, whereas bct is the blockchain process time, which must be added with the data transaction validation. The objective of the study was determined in the following way.

(12) $$T=\sum_{i=1}^{I}l_{i}^{e}+f_{i}^{e}+c_{i}^{e}+b_{i}^{e}+T_{i}^{e}$$

(13) $$\text{Cost}=\sum_{i=1}^{I}(l_{i}^{e}+f_{i}^{e}+c_{i}^{e}+b_{i}^{e}+T_{i}^{e})\cdot c_{i}^{e}$$

Equation (12) determines the total transaction delays on different nodes, while Equation (13) determines the total processing cost on different nodes.

## 4. Proposed Algorithm Methodology

The proposed algorithm methodology has three parts. In the first part, we show the entire algorithm flow as shown in Algorithm 1. In the second part, we show the local client socket-based blockchain and sandbox static and dynamic analysis execution flow on the local mobile devices. In the third part, we show the execution flow of the client socket-based blockchain and sandbox static and dynamic analysis for offloaded healthcare tasks in fog and cloud nodes during execution.
**Algorithm 1:** RBEFInput: *M*, *I*, *K*, *B*, *BC*, Ransomware-Trained [List] Output: Minimized *T,Cost*. **For** (*I* = 1 to *I*) **For** (*k* = 1 to *K*) Call Algorithm 2 to process the local healthcare tasks based on blockchain sandbox scheme Call Algorithm 3 to process the fog cloud healthcare tasks based on blockchain sandbox scheme **End Main Assignment** **End Main Loop**

The main goal of Algorithm 1 is to find the best transaction between the different nodes in the system, which are based on the blockchain. Based on the rules of Kotlin, each blockchain validation has a socket client and server. All workloads are registered based on smart-contract rules to keep people from being anonymous on the network. Initially, all workloads are encrypted based on the blockchain and offloaded to fog and cloud nodes via base stations for processing. Our goal is to optimize the objective as given in the algorithm, e.g., =$m_{i}^{e}+f_{i}^{e}+c_{i}^{e}$ + $b_{i}^{e}$. Algorithm 1 executed all transactions with the minimum execution cost and storage cost in the client–server architecture. 

Algorithm 2 is the client blockchain sandbox scheme, where all healthcare tasks are preformed locally and offloaded to the socket server for further processing. Each task must be encrypted based on SHA-256 in the particular blockchain block. Each transaction has particular pattern of hashing, which is generated randomly based on basing characterization. The main reason of random pattern is that the random pattern is more secure and non-repetitive. The static analysis process starts automatically when the offloading and downloading transactions exceed the time limit. If the transaction time limit is exceeded, the previous block transaction and current blockchain automatically start matching again. If the blockchain blocks are delayed and not matched, the algorithm automatically searches for them from the static analysis-trained list. However, if the algorithm does not find a ransomware attack from the analysis list, and blockchain hashing is not matched, it starts the dynamic analysis and matches the new-found pattern with the original one and adds the new pattern as an unknown pattern in the list.
**Algorithm 2:** Client Blockchain Sandbox SchemeInput: *M*, *I*, *B*, *BC*, Ransomware-Trained [List] Output: Minimized *T*. Sandbox Sampling Trained [List] **For** (*I* = 1 to *I*) Determined assignment the local execution delay based on Equations (1)–(3) and (8) Determined the local blockchain at mobile wallet blockchain validation based on Equation (4) Apply the Static Analysis **If** (*m*~*i*~*bc*1~Ransomware-Trained [List]! = success) { Apply the blockchain hashing *AES*-256 to workload *i* based on Equations (5)–(7) All transactions must have blockchain pattern (Random hashing and time) } **If** (*b*1~*i*~*bc*2~*m*1~Ransomware-Trained [List] = success) { Call Dynamic Analysis Determined the pattern~*b*1~*i*~*bc*1~Ransomware-Trained [List] Repeat Until ~Ransomware-Trained [List]! = success Matched the pervious hash *b*1~*i*~*bc*1~Ransomware-Trained [List] = success Apply the blockchain hashing *AES*-256 to workload *i* All transactions must have blockchain pattern } **End Main Assignment**
**End Main Loop**

Algorithm 3 also determines the processing time and processing cost locally before offloading healthcare tasks to the socket server based on distributed healthcare system. This approach enhances the security and efficiency of the mobile healthcare system, enabling patients to offload and download various healthcare services for their usage.
**Algorithm 3:** Server Blockchain Sandbox SchemeInput: *M*, *K*, *B*, *BC*, Ransomware-Trained [List] Output: Minimized *T*. Sandbox Sampling Trained [List] **For** (*k* = 1 to *K*) Determined assignment the fog cloud execution delay based on Equation (10) Apply the Static Analysis **If** (*m*~*i*~*bc*1~Ransomware-Trained [List]! = success) { Apply the blockchain hashing *AES*-256 to workload *i* All transactions must have blockchain pattern (Random hashing and time) } **If** (*k*1~*i*~*bc*2~*k*2~Ransomware-Trained [List]! = success) { Matched the pervious hash *k*1~*i*~*k*2~Ransomware-Trained [List]! = success Apply the blockchain hashing *AES*-256 to workload *i* All transactions must have blockchain pattern Determine the cost based on Equations (12) and (13) } **If** (*k*2~*i*~*bc*2~*k3*~Ransomware-Trained [List] = success) { Call Dynamic Analysis Determined the pattern~*k2*~*i*~*k3*~Ransomware-Trained [List] Repeat Until ~Ransomware-Trained [List]! = success Matched the pervious hash *k2*~*i*~*k3*~Ransomware-Trained [List] = success Apply the blockchain hashing *AES*-256 to workload *i* All transactions must have blockchain pattern } **End Main Assignment** **End Main Loop**

Algorithm 3 is the client blockchain sandbox scheme between the fog and cloud nodes. All healthcare tasks are performed on different fog and cloud nodes. Each task must be encrypted based on SHA-256 in the particular blockchain block. Each transaction has particular pattern of hashing, which is generated randomly based on basing characterization. The main reason of random pattern is that the random pattern is more secure and non-repetitive. The static analysis process starts automatically when the offloading and downloading transactions exceeds the time limit. If the transaction time limit is exceeded, the previous block transaction and current blockchain automatically start matching again. If the blockchain blocks are delayed and not matched, the algorithm automatically searches for them from the static analysis-trained list. However, if the algorithm does not find a ransomware attack from the analysis list and blockchain hashing is not matched, It starts the dynamic analysis and matches the new-found pattern with the original one and adds the new pattern as an unknown pattern in the list.

All the fog nodes are connected to each other, where all transactions of blockchain-based transactions are validated during execution and shared between hospitals.

## 5. Performance Evaluation and Implementation

In this session, the study shows the designed simulator and discusses the result with the existing blockchain schemes in the framework. The simulator parameters are defined in the Table 3.

### RBEF Implemented Simulator

**Figure 2:**
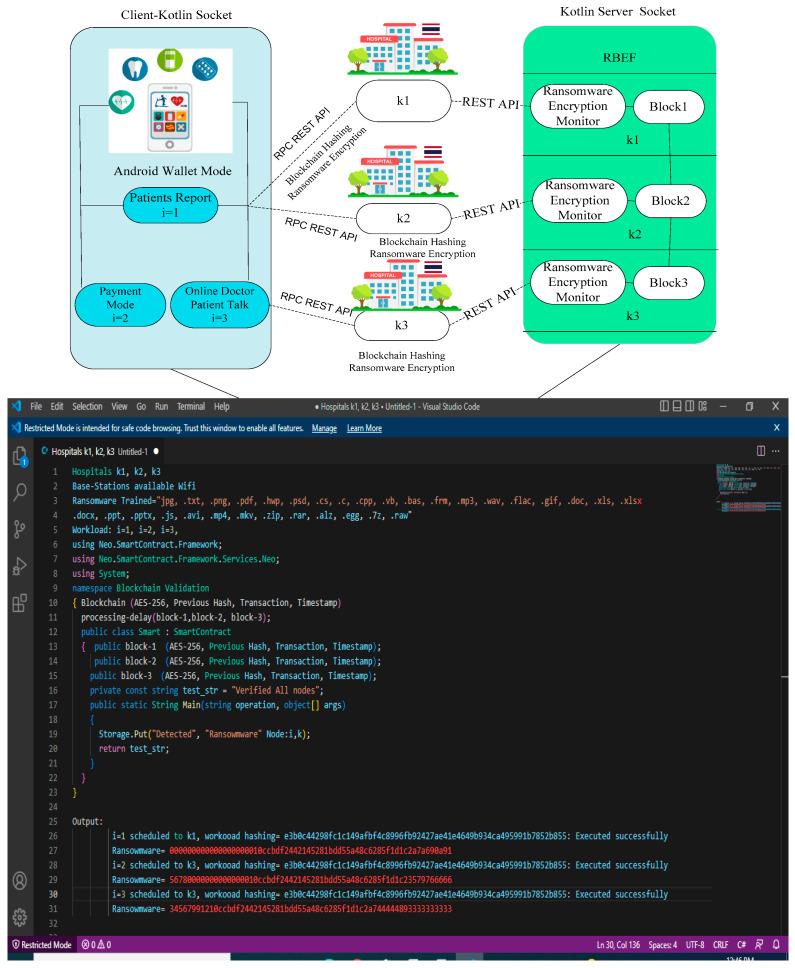
Implemented RBEF Based on Android Kotlin RPC Socket Programming.

The study designed the RBEF simulator based on Android Kotlin RPC for healthcare applications, as shown in Figure 2. The simulator consists of different work-loads, such as report monitoring, payment mode, and remote online patient and doctor monitoring services. The Android healthcare wallet mode has the ransomware monitoring and smart-contract rules in which blockchain hashing and ransomware hashing are verified and predicated based on the RBEF schemes, as shown in Algorithm 1 on the different nodes.

**Figure 3:**
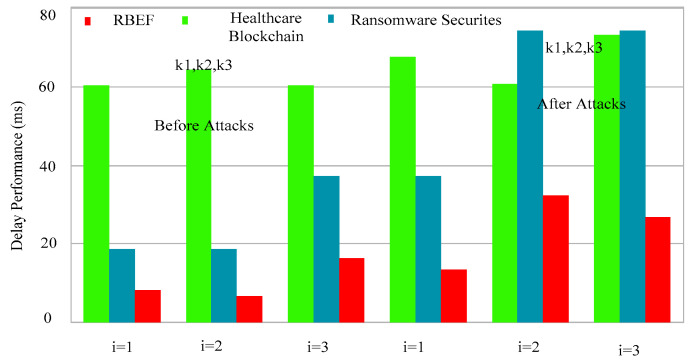
Delay T and Ransomware Performances of RBEF and Existing Blockchain Schemes on Distinct Tasks.

There are different nodes in the RBEF, such as mobile nodes, wireless nodes, and blockchain fog cloud nodes, which are connected via different REST API based on JSON protocols in the framework. Each data transaction among nodes must be identified by blockchain hashing and ransomware hashing to prevent any attacks on the data. Each node has blockchain blocks and a ransomware-trained list to determine the any random attacks in the system. Generally, each blockchain block serves as a miner that has capability to perform transform transactions in the network. Figure 2 illustrates the framework, where all nodes are implemented, the ransomware-trained list is embedded, and blockchain hashing and smart-contract rules are enforced. At the end, the red line in the figure represents ransomware attacks detected through hashing, distinct from blockchain attacks within the framework. Figure 3 shows that the proposed RBEF framework outperformed all existing ransomware techniques [1,2,3,4,5] and blockchain schemes [7,8,9,10,11,12,13] for healthcare application in terms of delay and the ability to identify the ransomware attacks across the different nodes.

In our simulator, hospitals have been added as partners based on blockchain technology. The hospitals are added based on preconditions (e.g., all data must be encrypted and decrypted in the blocks and verified data based on proof of work schemes). For instance, Hospital 1 is added to the node k1; however, after adding the hospital in the system, it can share the data with other hospitals, such as k2 and k3. The system is designed based on socket programming. If the one hospital updates or deletes some distinct records, it must inform all connected partners during the processing in the system. As a result, all hospitals share encrypted and valid data with each other. Additionally, each hospital has the capability to encrypt and decrypt data within their respective blockchain blocks in the network.

The impact of cyberattacks, such as ransomware attacks, on the transactional delays in blockchain technology is demonstrated in the table. The study analyzed attacks on socket programming-based blockchain technologies in remote procedure call (RPC) at various times and across different time zones, longitudes, and latitudes due to distributed geographical locations. Different types of attacks were analyzed during the execution of healthcare workloads on socket blockchain technology, including known ransomware attacks, such as Ryuk and SamSam, as well as unknown attacks.

During the experiments, it was observed that these attacks prevented transactions from executing on different nodes, resulting in longer delays that caused missed workload deadlines. One of the main issues observed was that the required resources were exploited more than the initial requirements, as they consumed more resources during recovery from cyberattacks. Table 4 shows that workloads or data, such as i = 1 and i = 2, incurred additional transactional delays. For example, during the processing of healthcare workloads in the fog cloud networks, 5, 3, 7, 6, 4, 5, and 10 transactional delays were observed.

Table 5 presents the performance of different suggested blockchain-enabled methods, along with the proposed work for healthcare workloads, in response to ransomware attacks. Method BSFR-SH was attacked with Ryuk on different nodes, namely i = 1, i = 2, and i = 3, implemented between 50, 100, and 500. The assigned deadlines and processing delays for all nodes were 18, 20, 25, and 30, 30, 40, respectively. The study analyzed the attacked workloads i = 1, i = 2, and i = 3, with transactional delays of 12, 10, and 15, respectively. The longitude and latitude show the impact of cyberattacks on the resources. For instance, the longitude in Table 4 shows the ransomware attacks degraded the performances of healthcare application with the different range of levels. For instance, values of 51.4, 45.4, 33.9, 40.9, 45.8, 42, and 40 were obtained with the different number of ransomware attacks in the distributed healthcare computing nodes during execution of tasks. The latitude shows that the accessibility of resources and services on different healthcare nodes could suffer in negative way: for instance, −87, −65, −67, −78, −89, −83, and −81. These values show that the access of healthcare services could be busy due to ransomware attacks; the normal range is 0.1 and 1. However, we only discussed the maximum failure range of nodes during their attacks for healthcare tasks. 

The study also analyzed IPFS Ryuk on different nodes i = 1, i = 2, and i = 3, implemented between 55 and 100, with deadlines of 16, 20, and 25, and total delays of 25, 30, and 36, respectively. Transactional delays were 9, 10, and 11, with affected workloads being i = 1 and i = 3 during the experiment with this method. The remaining methods are explained in Table 5. However, the proposed method, RBEF, showed lower transaction delays and only missed the deadline of i = 1. The RBEF system was trained using various ransomware sampling attacks with different features and classifications, as shown in the following link: https://www.kaggle.com/datasets/samara2022/ransomware-attacks (accessed on 21 September 2022). The static and dynamic analysis schemes are integrated to identify the ransomware attacks in healthcare application. We integrated the https://cuckoosandbox.org/download (accessed on 21 September 2022) API, which is publicly available for usage. The static analysis scheme consisted of 1500 ransomware attacks patterns: for instance, Ryuk, conti, SamSam, and others. The dynamic analysis scheme added as the unknown ransomware attack in the blockchain based on the healthcare application during execution in the distributed mobile fog cloud networks.

## 6. Results and Discussion

### 6.1. Results and Discussion of Performance on Dynamic Analysis of Ransomware Attacks

The analysis of blockchain technologies in terms of security processing, validation, and data transaction delays among nodes was performed in this study. Various nodes were connected within the blockchain network, and all nodes were heterogeneous in nature. The study utilized different socket-based blockchain technologies, such as healthcare blockchain [11,12,13,14,15,16,17,18,19,20] and ransomware-enabled blockchain technologies [20,21,22,23,24,25,26,27,28], as baseline approaches. The RBEF scheme was developed to evaluate the performances of healthcare workloads compared to existing studies, considering delay constraints within the system.

Figure 3 demonstrates the significant impact of ransomware attacks on the performance, resulting in increased local processing delays for distinct tasks. Existing blockchain technologies only verified the transactions among nodes; however, attacks on transactions, particularly ransomware attacks, can significantly degrade the performance of blockchain in healthcare applications. The blockchain blocks consist of different transactions that are sequentially validated, leading to long delays when local, network, and different fog and cloud networks are interconnected.

**Figure 4:**
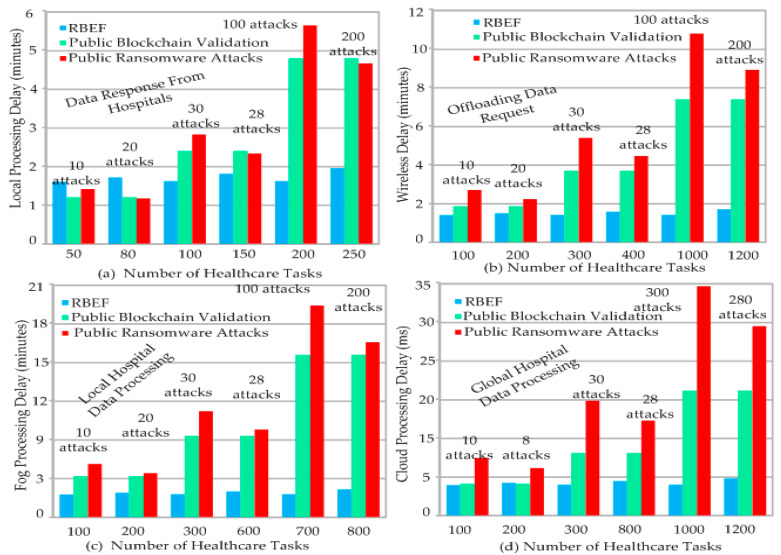
Different Processing Delays in Minutes at Different Computing Nodes with Different Attacks.

The study analyzed the blockchain performances on the blockchain nodes based on and attacks and hashing with the different hashing tasks, attacks, and outputs with the different nodes. Cyberattacks can interfere in the system. The study implemented different blockchain frameworks such as ransomware-efficient blockchain [27,28,29,30]. Figure 4 shows the blockchain performances of different nodes such as local processing delay, network delay, and fog and cloud delays. Figure 4a shows the performance of healthcare tasks, such as 50, 80, 100, 150, 200, and 250. We attempted different ransomware attacks on tasks, such as 10, 20, 30, 28, 100, and 200. Initially, RBEF took 2 min compared to public blockchain and ransomware techniques. The main reason is that we initially added the sandbox static and dynamic schemes inside the blockchain validation. Therefore, it takes more time at the start. However, after 100, 150, 200, and 250 attacks on blockchain blocks, the existing blockchain technologies have higher local processing delays, about 5 min, compared to RBEF. The main reason is that the current technologies exceeded the transactional time limits during attacks, and for extra validation, they took more time. However, RBEF controlled all attempts during and after execution on the different mobile devices. Figure 4b shows the communication processing delays during offloading and downloading of data. The number of healthcare tasks, such as 50, 80, 100, 150, 200, and 250, and attempted ransomware attacks (10, 20, 30, 28, 100, and 200) with RBEF had a lower wireless delay than existing techniques. The main reason is that RBEF divided the application into client and server socket sides, where few transactions suffered attacks. However, existing blockchain technologies executed the entire block as coarse-grained when the ransomware attempts were applied to the block; due to many tasks on the same node or network, it incurred higher delays. Figure 4c shows the processing delays of offloaded tasks on the different local fog node-enabled servers. The number of healthcare tasks, such as 50, 80, 100, 150, 200, and 250, and attempted ransomware attacks (10, 20, 30, 28, 100, and 200) with RBEF had a lower wireless delay than existing techniques. The main reason is that RBEF is divided among different fog nodes. Therefore, attacks on distinct fog nodes can be easily recovered, and patterns are matched with a lower processing delay. However, existing blockchain technologies executed the entire block as coarse-grained when the ransomware attempts were applied to the block; due to many tasks on the same node or network, it incurred higher delays. Figure 4d shows the processing delays at cloud computing during data storage for saving and downloading executed tasks. The number of healthcare tasks, such as 50, 80, 100, 150, 200, and 250, and attempted ransomware attacks (10, 20, 30, 28, 100, and 200) with RBEF had a lower wireless delay than existing techniques. The main reason is that RBEF is divided among fog nodes and cloud servers. Therefore, attacks on distinct fog nodes can be easily recovered, and patterns are matched with a lower processing delay. However, existing blockchain technologies executed the entire block as coarse-grained when ransomware was applied to the block. Due to many tasks on the same node or network, it incurred higher delays.

Due to this, the delays in the different nodes increased and minimized the system performance. Hence, it is necessary to improve the performance of the blockchain in terms of cyberattacks along with the verification and transactions validation in the system. Figure 4 shows the proposed work RBEF algorithm as compared to public blockchain and ransomware and blockchain with non-dominated schemes in terms of resource leakage. The main reason of the resource leakage is from the huge process of the blockchain that required a lot of resources in the system.

Figure 4a evaluates the performances of local devices and healthcare services downloaded from hospitals. We executed different tasks in a range of 50 to 250. For instance, healthcare patient registration, report downloading, patient login, appointment scheduling, and other services were executed on local devices. We attempted the different attacks on the local devices and analyzed the performance of the algorithms. We attempted 10 ransomware attacks on 50 tasks and then analyzed the method’s performance to detect and process them in architecture. In this case, we applied the static analysis technique and blockchain technologies. However, existing blockchain technologies ignored these perspectives, and we saw that they incurred higher local processing delays on mobile devices. We attempted 250 attacks on different tasks and observed the methods’ performances. However, ransomware-efficient and generalized blockchain technologies incurred a higher delay in minutes. We implemented the RBEF validation at different phases, such as mobile, wireless, fog, and cloud. Therefore, RBEF obtained optimal results as compared to existing blockchain technologies. Figure 4b shows the offloading of data requests or services from mobile devices to the cloud through wireless networks. We attempted 200 ransomware cyberattacks on 1200 requests. We observed that the blockchain validated invalid transactions in terms of services in the networking phase, which is the communication between the cloud and mobile devices. It is observed that blockchain technologies do not allow for capturing data, but validating and identifying invalid transactions takes a lot of time in different transaction blocks in their architectures. Figure 4c shows the performance of blockchain technology when healthcare handles the local mobile patient tasks and processes them according to the given request on the fog servers. Figure 4d shows the blockchain check validations and processes on different tasks in the cloud server that are geographically distributed in the global networks. For instance, in our case, all the local metropolitan hospitals are connected with the global hospital in the country where these systems will be applied. It is observed that the delay is an essential factor, which was widely ignored in previous blockchain technologies when they suffered from ransomware cyberattacks in the healthcare system. Therefore, RBEF obtained optimal results regarding transactional delays across different networks. The main reason is that we monitored both the static and dynamic analysis of ransomware attacks in advance on different nodes instead of mobile patients’ wallet data and cloud hospital data. In this way, we can control transaction delays and failure ratios in blockchain-enabled healthcare systems.

### 6.2. Results and Discussion of Performance on Dynamic Analysis of Ransomware Attacks

In this part, we discuss runtime ransomware attacks that attempt to encrypt the patients’ healthcare files at different nodes. Generally, we implemented the sandbox API to identify the pattern and functioning of the ransomware attacks on the distributed nodes. The sandbox API offers different sampling data for ransomware, so we can efficiently identify its pattern and behavior. Therefore, in this part, we discuss and identify the performance of blockchain methods for healthcare applications.

**Figure 5:**
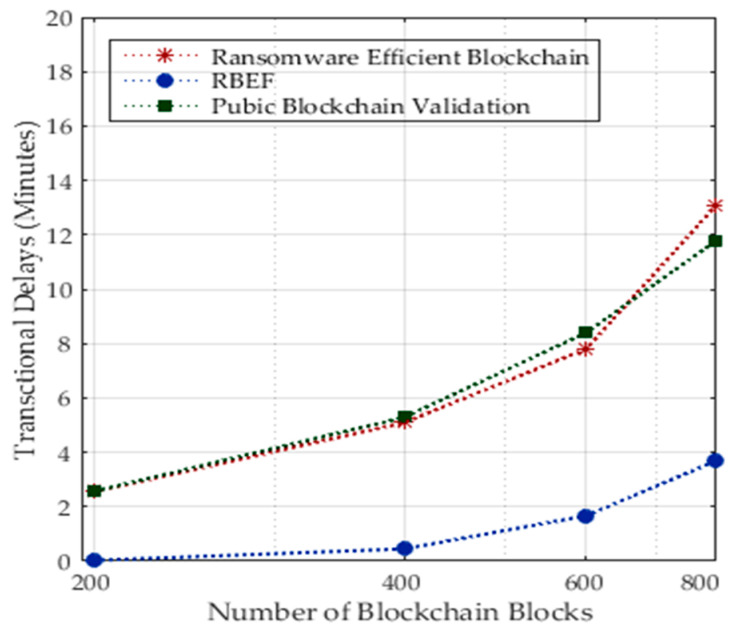
Transaction Delays in Blockchain Blocks during Runtime Ransomware Attacks.

Dynamic analysis was employed to identify ransomware attacks on distributed nodes. These ransomware attacks were not trained and tested using the designed methods before the healthcare systems were implemented. Therefore, these types of ransomware attacks were identified as unknown attacks on data transactions in healthcare systems. Figure 5 shows the transactional delays due to dynamic ransomware attacks on different nodes. RBEF performed well with all existing blockchain schemes in terms of delays. The main reason is that all the existing schemes made all detections on a single node. Therefore, it is a time-consuming process, and the ratio of processing delays in transactions and failure ratio increase.

**Figure 6:**
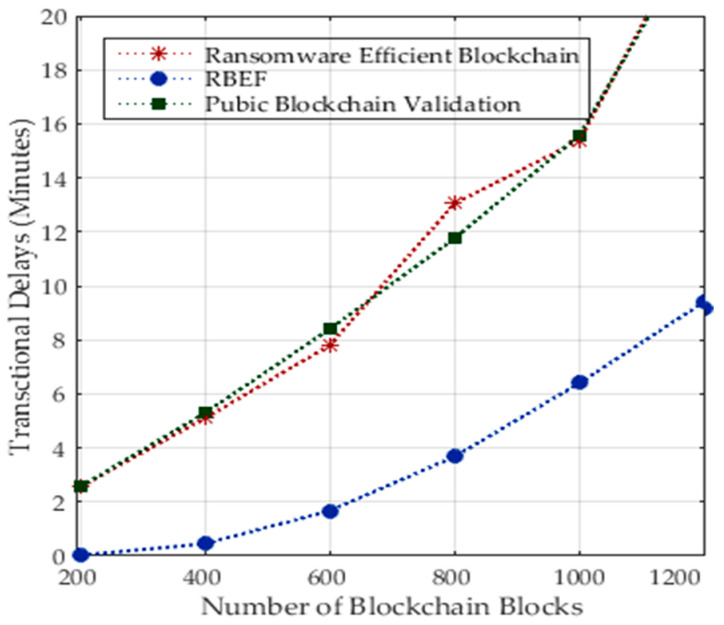
Transaction Delay during Runtime Ransomware Attacks in Blockchain Healthcare Transactions.

It is evident from Figure 6 that increasing the number of transactions across different healthcare nodes leads to higher processing delays when identifying the patterns of ransomware attacks that already exist in the files in the form of binary code.

**Figure 7:**
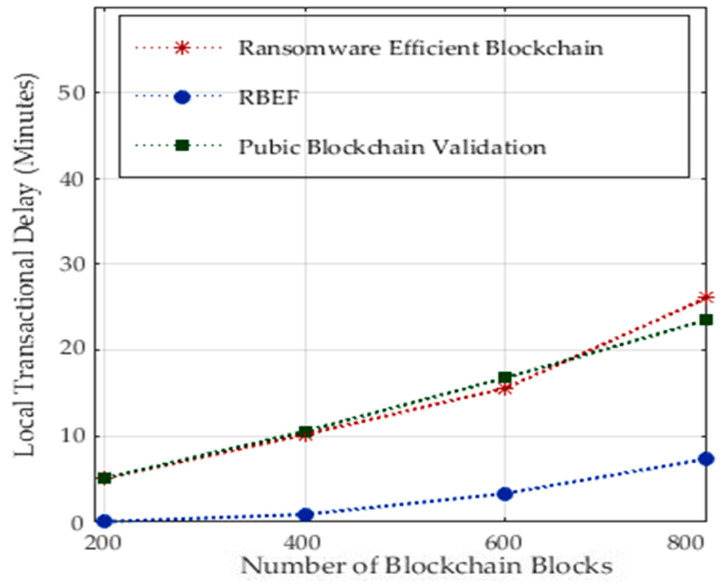
Mobile Dynamic and Static Analysis Blockchain Transactional Performance.

We implemented blockchain technologies at different nodes and analyzed the performance of each processing node during distributed healthcare applications. In healthcare architectures, socket programming divides applications into patient devices and hospital nodes for processing, offloading, and downloading the data. Figure 7 shows the performance of local transactional delays on mobile devices during the execution of 800 healthcare tasks. RBEF implemented sandbox static and dynamic analysis for ransomware prediction at compile-time and runtime regarding transactional delays. Therefore, we developed delay optimal strategies and designed a lightweight socket RPC that divides the application between local and remote tasks. Only lightweight tasks are executed on mobile devices, such as login, registration, and downloading and offloading medical data. However, under their preset resources, local devices still encrypt and decrypt data based on blockchain schemes, including static and dynamic ransomware detections. However, existing public and ransomware-efficient blockchain technologies did not design this mechanism and executed all the transactions on distinct nodes. Therefore, they incurred higher processing delays as compared to RBEF.

**Figure 8:**
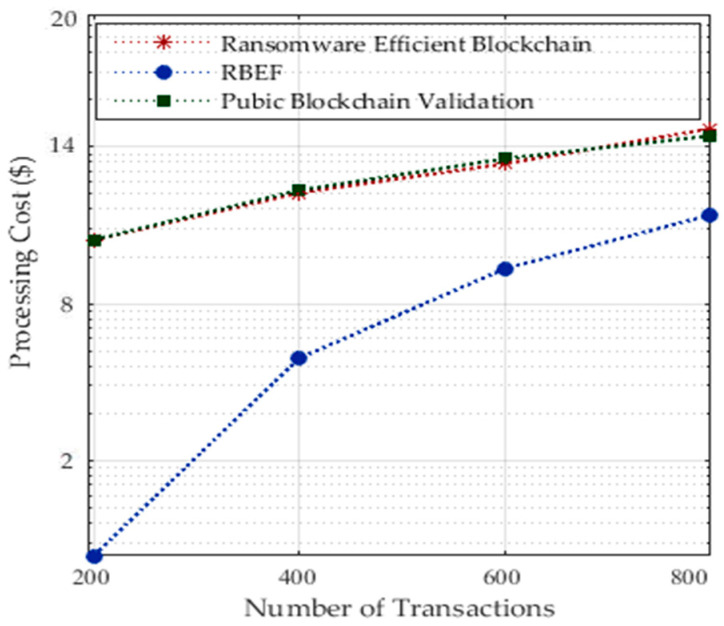
Processing Transactional Cost for a Different Number of Transactions.

In our architecture, we are only calculating the transactional cost in terms of renting services and processing tasks with the storage in a blockchain-enabled healthcare system. For instance, in socket RPC blockchain healthcare, all services are paid; you need to pay for data processing and storing patient’s data on the healthcare nodes. Figure 8 shows the processing of random number of 800 tasks with the required services incurred with the cost. However, according to cost for processing, we divided the application into local and remote processing in order to reduce cost as shown in Figure 8. RBEF incurred a lower processing cost as compared to existing blockchain technologies. In our architecture, we did not save all tasks’ data except the necessary data on the storage from the local socket to the server socket. In this way, we can minimize the cost. However, existing blockchain technologies only focused on security constraints; they widely ignored the processing cost in their technologies. Each healthcare application charged for security services in terms of scanning before downloading and scanning during execution of files inside mobile devices. Therefore, new technologies must be cost-efficient as we showed in our architecture for healthcare tasks.

**Figure 9:**
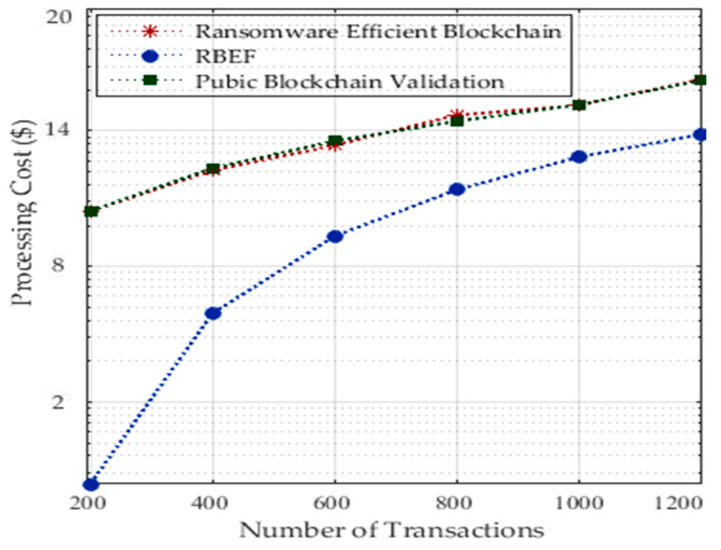
Processing Cost and Storage Cost at Hospital Nodes.

Figure 9 shows the processing of a random number of 1200 tasks with the associated costs for each task. However, to minimize costs, we divided the application into local and remote processing, as depicted in Figure 8. RBEF demonstrates lower processing costs compared to existing blockchain technologies. In our architecture, we implemented a strategy where only necessary task data are stored on the storage from the local socket to the server socket, allowing us to reduce costs. However, existing blockchain technologies have primarily focused on security constraints while widely disregarding the processing costs associated with their technologies. The dynamic and static analysis of ransomware attacks for applications incurs processing costs in terms of security considerations.

**Figure 10:**
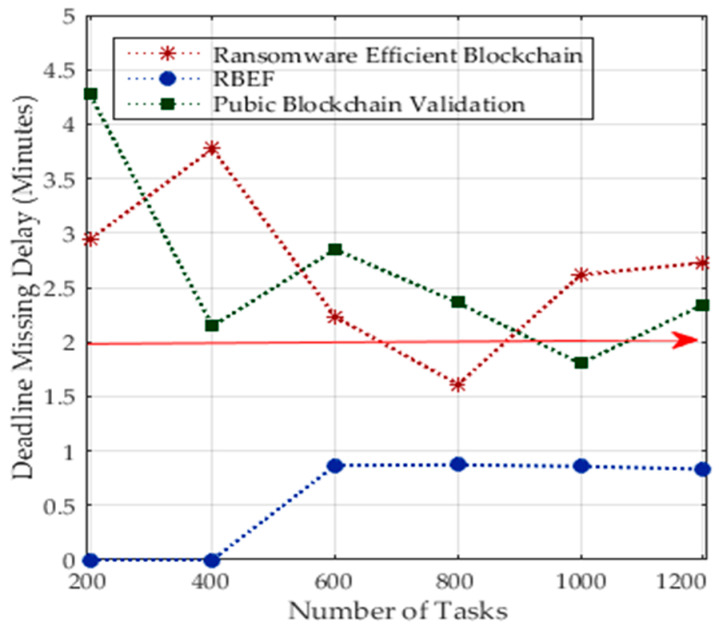
Deadline Performances of 1200 Healthcare Tasks.

All healthcare tasks have deadlines in order to be executed with the minimum cost and delay in the distributed networks. We analyzed the performance of healthcare tasks during execution in terms of static, dynamic, delay, and processing costs. Figure 10 shows public blockchain validations have a higher failure rate than deadlines and the threshold increased more than the preset. Due to ransomware detection with static and dynamic methods, it incurred higher processing costs and delays. RBEF outperformed existing blockchain validation and ransomware blockchain technologies. RBEF executed all healthcare tasks within their deadlines. The main reason is that we set all deadlines after scheduling tasks on the processing nodes. The existing blockchain technologies do not consider these aspects for healthcare tasks.

## 7. Conclusions

The paper presented the RBEF architecture and research methodology to process secure and valid data among healthcare nodes. The study demonstrated cuckoo sandbox-enabled static and dynamic analysis schemes at client and server sockets. We showed the simulation environment and data regarding the implementation of RBEF. The study discussed the objectives of the research and the advantages of the analysis of the results. We discussed the simulation results with the existing technologies and proposed work and analyzed the execution delays and processing costs for all healthcare tasks. We discussed the local processing delay, base station delay, and fog and cloud delay with all blockchain technologies when they are implemented in simulation environments. It can be observed from the simulation results that the proposed RBEF is more optimal for handling all kinds of delayed performances and transactions that existing blockchain technologies do not consider for healthcare tasks. The number of healthcare tasks, such as 50, 80, 100, 150, 200, and 250, and attempted ransomware attacks (10, 20, 30, 28, 100, and 200) with RBEF had a lower wireless delay than existing techniques. The main reason is that RBEF is divided among fog nodes and cloud servers. Therefore, attacks on distinct fog nodes can be easily recovered, and patterns are matched with a lower processing delay. We proved that the efficiency of RPC and socket-based RBEF minimized the processing delays between 2 and 10 min at different nodes and the processing cost decreased by 10% during transactions as compared to existing studies.

In future work, we will add the new constraints in RBEF, such as energy consumption and electricity cost, during real-time healthcare monitoring for critical patients with the hybrid data.

## Figures and Tables

**Table 1:** Existing Ransomware-Enabled Blockchain Schemes for Healthcare Systems.

Study	Security Scheme	Protocol	Attacks	Applications	Research Gap
[1]	Blockchain PoW	HTTP, FTP	Ransomware and data attacks	Healthcare	Higher delays
[2]	Blockchain PoW	TCP/IP, UDP	Static Ransomware Attacks	Healthcare	Higher delays
[3,4,5]	Blockchain PoC	TCP/IP, UDP	Static Ransomware attacks	Healthcare	Higher delays
[6,7,8]	Blockchain Semantic	TCP/IP, UDP	Anonymous attacks	Healthcare	Higher delays
[9,10,11,12,13,14,15]	Blockchain-enabled security	TCP/IP, UDP	Invalid transaction, failure node	Healthcare	Higher delays
[16,17,18,19,20,21,22]	Network Security	TCP/IP	Malware	Healthcare	Higher delays
[23,24,25]	Static Analysis	REST API	Known Ransomware	Healthcare	Higher delays
[26,27,28,29,30,31]	Dynamic Analysis	REST API	Unknown Ransomware	Healthcare	Higher delays
proposed	RBEF	RPC	Ransomware attacks	Healthcare	delays, cost

**Table 2:** Mathematical Notations.

Symbol	Description
I	Number of Healthcare Tasks
$i^{th}$	Particular task of healthcare
W	Number of workloads
$w_{i}$	Workload of task i
$d_{i}$	Deadline of workload i
K,M	Fog, Cloud, and Mobile Devices
k,m	Particular fog cloud and mobile
ζk	Speed of computing node
ζm	Speed of mobile devices
*B*	Number of base stations
b	Base station in network
x{i,b,k}= 0, 1, 2	Assignment of workload to the base station and cloud computing
BC	Number of blockchain blocks
bc	Blockchain block
h,i,k	Hashing attributes of blockchain
PK	Public key of the blockchain
PV	Private key of the blockchain
$L_{i}^{e}$	Local execution Delay
$b_{i}^{e}$	Downloading time
$T_{i}^{e}$	Offloading time
T	Total Delay
Cost	Total Transactional cost
$f_{i}^{e}$	Fog and Cloud Delay
$c_{i}^{e}$	Processing Cost

**Table 3:** Simulator Parameters.

Parameter	Value
Workload:i=1,i=2,i=3	https://physionet.org/ (accessed on 21 September 2022)
Platform	Android JAVA, KOTLIN
Runtime Environment	Android X86
k1, k2, k3	500 GB, 64 RAM, Core i7
m1	Android Appo-15, RAM 6G, Processing core
Blockchain Attributes	Public, AES-256,
Ransomware	Trained 500 Samples: https://github.com/ABDULLAH-RAZA/Heartbeat-Malware\_Detection-Dataset (accessed on 24 May 2021)
Upload and Download Bandwidth	Wifi: 20–56 mbps, heterogeneous

**Table 4:** Blockchain Hashing Against Ransomware Attacks.

Date Time	Attack	Layer	Industry	Longitude	Latitude	Effected Data	Transaction Delays (min)
1 January 2023 11.00 a.m.	Ryuk	Request RPC	Healthcare	51.4	−87	i = 1	5
5 January 2023 10.00 a.m.	Ryuk	Request RPC	Healthcare	45.4	−65	i = 1	3
5 January 2023 06.00 a.m.	unknown	Request RPC	Healthcare	33.9	−67	i = 1	7
9 January 2023 09.00 a.m.	Conti	Respond RPC	Healthcare	40.9	−78	i = 1	6
9 January 2023 10.00 a.m.	SamSam	Respond RPC	Healthcare	45.8	−89	i = 1	4
11 January 2023 10.00 a.m.	Unknown	Respond RPC	Healthcare	42	−83	i = 1	5
12 January 2023 10.00 a.m.	Ryuk	Respond RPC	Healthcare	40	−81	i = 2	10

**Table 5:** Existing Blockchain Architectures for Healthcare Workloads.

Blockchain	Attack	Workloads	Nodes	Deadline (min)	$T\;=\;l_{i}^{e}+f_{i}^{e}+c_{i}^{e}$ $+b_{i}^{e}$	Effected Data	Transaction Delays (min)
BSFR-SH [2]	Ryuk	i = 1, i = 2, i = 3	50, 100, 500	18, 20, 25	30, 30, 40	i = 1, i = 2, i = 3	12, 10, 15
IPFS [3]	Ryuk	i = 1, i = 2, i = 3	55, 100	16, 20, 25	25, 30, 36	i = 1, i = 3	9, 10, 11
Ransomware Blockchain [4]	unknown	i = 1, i = 2, i = 3	100, 250	19, 23, 21	27, 33, 31	i = 1, i = 3	8, 10, 10
Ransomware Blockchain [5]	Conti	i = 1, i = 2, i = 3	250, 300	15, 16, 19	35, 32, 38	i = 1, i = 3	20, 16, 19
FBASHI [13]	SamSam	i = 1, i = 2, i = 3	300, 500	13, 15, 17	31, 30, 34	i = 1, i = 2	18, 15, 17
Block MedCare [14]	Uknown	i = 1, i = 2, i = 3	500, 1000	15, 16, 18	33, 32, 36	i = 1, i = 3	18, 16, 18
Blockchain-IoMT [15]	Ryuk	i = 1, i = 2, i = 3	1000	14, 20, 25	27, 40, 50	i = 2, i = 3	13, 20, 25
Proposed RBEF	Ryuk, Conti	i = 1, i = 2, i = 3	1000	14, 20, 25	15, 19, 24	i = 1, i = 2, i = 3	10, 10, 10

## Data Availability

The ransomware sampling attacks data used in this study is available at https://www.kaggle.com/datasets/samara2022/ransomware-attacks, accessed on 21 September 2022.The integrated static and dynamic analysis schemes API is publicly available for usage at https://cuckoosandbox.org/download, accessed on 21 September 2022.
